# Health benefits of fish and fish by-products—a nutritional and functional perspective

**DOI:** 10.3389/fnut.2025.1564315

**Published:** 2025-05-09

**Authors:** Sana Noreen, Bushra Hashmi, Patrick Maduabuchi Aja, Ayomide Victor Atoki

**Affiliations:** ^1^University Institute of Diet and Nutritional Sciences, The University of Lahore, Lahore, Pakistan; ^2^Faculty of Biomedical Sciences, Kampala International University, Western Campus, Bushenyi, Uganda

**Keywords:** fish, fish by-product, polyunsaturated fatty acids, bioactive compounds, health benefits and risks

## Abstract

Fish is the primary marine source that provides adequate nutrition to the human body. Fish production is increasing every year, contributing to a sustainable economy, as they provide a significant source of income and food: This review highlights the potential health benefits, industrial applications, and toxicity of various fish species Globally, fish possess various bioactive compounds; this efficacy makes them a more edible source to consume worldwide. A wide range of bioactive compounds, primary macronutrients, micronutrients, vitamins, and minerals are present in various fish types that are essential in preventing different human disorders. The nutritional value of fish helps to provide exceptional health benefits against different human ailments. Fish are excellent sources of protein, peptides, and polyunsaturated fatty acids, particularly eicosapentaenoic acid (EPA), Alpha-linolenic acid (ALA), omega-6 fatty acids, and docosahexaenoic acid (DHA), omega-3 fatty acids that influence human health positively. Fish and their by-products are also excellent sources for developing various nutraceutical and functional foods to fight against multiple human disorders. The by-products of fish exert significant effects against infection, viral attack, cardiovascular diseases, immune disorders, oxidative stress, inflammation, neurological diseases, and other physiological complications. Few fish species are contaminated with harmful substances that cause potential risks to children's and adults' bodies. Additionally, the therapeutic use of fish and their by-products unveils the potential nutritional benefits to reduce the burden on public health by managing dietary issues such as food security, protein deficiency, and other nutritional-related problems.

## Introduction

Fish is an important source of high-quality protein, essential fatty acids (especially omega-3 fatty acid), vitamins, and minerals, making it vital for maintaining overall health, supporting brain function, cardiovascular health, and promoting growth and development, while also contributing to the prevention of chronic diseases such as heart disease, diabetes, and cognitive decline. Additionally, fish play a crucial role in food security and sustainable nutrition, especially in coastal and low-income regions. The chemical composition of raw fish comprises 0%−0.5% of carbohydrates, 16%−21% of protein, 1.2%−1.5% of minerals, 0.2%−25% of fat, 66%−81% of water, essential amino acids (EAA), and fatty acids. These nutritional components play a key role in promoting human health, supporting proper growth of children, preventing cardiovascular diseases, and protecting against other physiological health problems (1). Fish also provide essential nutrients, including omega-3 fatty acids, methionine, iron, zinc, vitamin A, vitamin D, lysine, and calcium. The content of fatty acids in fish varies in different species. In developing countries, fish fulfill the requirements of all essential nutrients and contribute to reducing food insecurity (2). Fish consumption contributes to lowering the public health burden by eradicating malnutrition (3). The metabolism and growth process in fish are regulated by growth hormones (GH) and insulin-like growth factors (IGH) that play a key role in the regulation of fish growth and metabolism (4). The metabolic processes in fish vary from species to species due to variations in temperature and environment. Various fluctuations affect the temperature in water and influence the physiological process, growth, and metabolism in fish (5). Different fish species and their by-products provide pharmaceutical, industrial, nutraceutical, and cosmeceutical properties due to the presence of collagen, enzymes, antioxidants, bioactive peptides, polyunsaturated fatty acids (PUFA), and other functional bioactive components (6). The world's aquatic ecosystem mainly comprises fish habitats that play a crucial role in aquatic food webs. Globally, fish play a key role as a good source of income for millions of people and the richest animal protein source (7). Around 8 billion people in the world consume fish. The rich amount of nutrients in fish helps to reduce the deficiency of various essential nutrients (8). A wide range of natural resources for fish and other aquatic life is present in the world. The production and exportation of fish have increased in recent decades (9, 10). Globally, around 32,000 fish species are documented by scientists. The production of fish plays a significant part in the global provision of food, aquaculture, and livestock industries (11–13). A few common fish species, such as *Labeo rohita, Cirrhinus mrigala, Hypophthalamichthys nobilis*, and *Cyprinus carpio*, are commonly found in other South Asian countries (14, 15). However, consuming contaminated fish poses harmful health risks due to various potential hazards, such as plastic waste and environmental contaminants. Major common contaminants in fish include selenium, polycyclic aromatic hydrocarbons (PAHs), polychlorinated biphenyls (PCBs), cadmium, arsenic, Methyl mercury, lead, dioxins, and chlorinated hydrocarbon pesticides. Generally, these contaminants enter the fish's body through the gills or skin. When consumed in large amounts, these contaminants build up in fish tissues and badly affect human health (16). Vulnerable groups such as pregnant women, children, and breastfeeding women are at high risk of being affected by fish contaminants. The fish contaminants, such as methylmercury, persistent organic pollutants, PCBs, and dioxins, cause negative effects on the fetus's neurological development and increase the risk of cognitive deficits and preterm birth. PCBs, Methyl mercury, and dioxins cause adverse health consequences in breastfeeding women, such as immune system dysfunctions, neurobehavioral impairments, cardiovascular disease, dementia, and cancer. A few species, including king mackerel, trout, tilefish, swordfish, and shark, are the most commonly contaminated fish. Vulnerable groups should avoid such contaminated fish and consume less contaminated fish, including catfish, salmon, cod, and tuna (16).

Climate changes, particularly sea-level rise, harmful algal blooms, water resources, and other diseases negatively impact on aquaculture production and reduce the total production of fish. Globally, a reduction in fish production and fluctuations in aquaculture exert adverse effects on the economy, nutraceutical industries, and the food system. There is a need to manage the environment of the aquatic ecosystem sustainably to protect aquaculture from harmful fluctuations of climate change (17). By 2025, aquaculture will be considered the chief aquatic dietary protein to meet the demand for food and a sustainable food supply (18). This article comprehensively analyzes fish, its by-products, nutraceutical applications, therapeutic benefits, and related problems. This article also emphasizes exploring the benefits of fish and their by-products against various chronic diseases. The article offers effective therapeutic potential to reduce diseases in a community and worldwide. The main aim of this review article is to focus on the nutritional value of fish, its bioactive compounds, fish-based by-products, and their therapeutic potential, while also addressing their role in managing and eradicating public health-related diseases.

## Chemical composition of fish and its by-products

A wide range of bioactive compounds, micro- and macronutrients present in fish and their by-products provide potential health benefits to human health. The nutritional profile and bioactive compounds in fish vary from species to species. Major bioactive compounds and vital nutrients in fish and their by-products are essential fatty acids, particularly omega-3 fatty acids, docosahexaenoic acid (DHA), alpha-linolenic acid (ALA), and eicosapentaenoic acid (EPA). The mineral composition of fish demonstrated includes essential vitamins such as dietary vitamins A, E, D, B3, B12, and B6 and several minerals such as selenium (Se), sodium (Na), iron (Fe), magnesium (Mg), iodine (I), zinc (Zn), calcium (Ca), and potassium (K) are present in fish (19, 20). The by-products of fish also contain multiple essential nutrients and bioactive components such as collagen, gelatin, Tocopherols, protein, carotenoids, bioactive peptides, chitin, phenols, tocotrienols, phytosterols, chitosan, and chitooligosaccharides that help to promote human health. The bioactive peptides and protein hydrolysates obtained from by-products of fish served as potential sources for producing functional food ingredients (6, 21). The by-products of fish, such as skin, muscle, and bone, contain antiaging, antimicrobial, wound healing, and anticoagulant components that contribute to the prevention of skin from microbes and other harmful substances as mentioned in Table 1. The bioactive peptides in by-products of fish provide nutritional and functional properties that play a vital role in preventing various diseases, including diabetes, cardiac disorders, and many other health problems (22, 23). The fish products provide various cosmetic, nutraceutical, and pharmaceutical applications. The phenolic compounds in fish and their by-products are also used in the food industry to manufacture dietary supplements. The therapeutic properties of various bioactive compounds in by-products of fish, such as carotenoids, help to inhibit stress-induced lipid peroxidation (24). The incorporation of nutraceuticals and fortification from by-products of fish helps to reduce the deficiency of nutrients and enhance the quality of life (25, 26).

**Table 1:** Applications of fish by-products.

**By-products of fish**	**Applications**	**References**
Gelatine	Gelatin is widely used for the development of various functional foods, pharmaceuticals, and nutraceuticals. It also contributes to providing various health benefits, including antihypertensive and antiosteoarthritis.	(28, 61)
Fish albumin and peptides	Fish protein and bioactive peptides are involved in many physiological functions, including suppression of angiotensin-I-converting enzyme (ACE) utilization of nutrients, immunomodulatory, anticoagulant, antioxidant, and antimicrobial properties.	(35)
Squalene	The extracted and purified squalene has immense biological properties, such as antioxidant and antihypertension. It also protects from pigmentation, inflammation, aging, oxidative stress, and other skin ailments.	(69)
Tuna eyes	The by-products of Tuna (*Thunnus thynnus*) such as eyeballs possess potential anti-inflammatory properties by inhibiting inflammatory cytokines such as nitric oxide. It also provides antioxidant activity against oxidative levels	(14)
Fish calcium	Fish bones are a huge source of calcium and phosphorus that play a vital role in providing health benefits and biomedical applications.	(75)
Shark cartilage	The cartilage provides prevention from osteoarthritis, inflammation, and obesity, and promotes anticoagulant activities. It enhances the modulation of intestinal microbiota and provides neuroprotective effects and bone regeneration activities.	(34)
Chitin and Chitosan	Chitin and chitosan play significant roles in multiple fields, including agriculture, biomedical, cosmetics, and food industries. The extracted chitin and chitosan also provide biotechnological applications in textile industries as the refinement of industrial effluents and chelating agents	(23)
Fish Oil	Fish oil provides physiological effects such as cardioprotective, antioxidant, neuroprotective, and anti-inflammatory effects. The supplementation of fish oil has gained significant interest in biomedical applications. It also possesses main importance in food industries for the development of nutraceuticals.	(21)
Fish meal	The fish meal comprises various essential vitamins such as cyanocobalamin (B12), pantothenic acid, niacin, riboflavin, and choline. The nutrients present in fishmeal are a great source of protein that provides potential to human health by promoting antioxidant and anti-inflammatory capability.	(71)

## Health benefits of fish and its by-products

The consumption of different species of edible fish and their by-products is of great interest in the field of nutrition due to multiple health benefits. The valuable nutritional profile of fish prevents various human diseases (27). Several essential nutrients, such as vitamin D, omega-3 fatty acids, selenium, potassium, and many other essential nutritional components, help to improve overall wellbeing as mentioned in Table 2. Few fish species, including shark, king mackerel, and swordfish, exert harmful effects on human health due to toxic contaminants present in these fish. The primary harmful fish contaminants are methylmercury, persistent organic pollutants, PCBs, and dioxins, which cause negative effects on human health. Methyl mercury and dioxins cause adverse health impacts in breastfeeding women, such as immune, cardiovascular disease, dementia, systemic dysfunctions, cancer, and neurobehavioral impairments, inflammatory, antioxidant, antiobesity, and antihypertensive properties (28). Fish are rich sources of brain-promoting nutrients such as omega-3 fatty acids, which help to improve blood flow in the brain, learning, cognitive behavior, and thinking, which prevents diseases like dementia. The nutritional composition of fish helps to enhance heart health and protect from stroke and other cardiac-related problems. The rich amount of protein in fish helps to reduce diabetes by improving insulin sensitivity and glycemic control. Some edible fish, such as *Salmo salar* (salmon) and *Thunnus thynnus* (tuna), are low in fat and protein and vitamin B12, which help to maintain good health. The adequate consumption of fish also promotes optimal gut health and provides numerous health benefits to gut microbes. Fish play an essential role in providing adequate nutrients and health benefits.

**Table 2:** Therapeutic function of different bioactive compounds derived from various fish species.

**Fish species**	**Bioactive compounds**	**Therapeutic action against different diseases**	**References**
Tuna (*Thunnus tonggol*), Atlantic salmon (*Salmo salar*), Tuna (*Thunnus* spp.), Catfish (*Clarias* spp.)	Peptides, protein, omega-3 PUFA, taurine, selenium, bioactive peptides	Antihypertensive, anti-inflammatory, cardioprotective, reduce serum triglyceride levels	(9)
Sardines (*Sardinella longiceps*), Atlantic salmon (*S. salar*), Herring (*Clupea harengus*), Tilapia (*Oreochromis* spp.), Eel (*Anguilla* spp.)	Omega-3 fatty acids, DHA, EPA, astaxanthin, peptides, protein	Antidiabetic, anti-inflammatory, antioxidant, promotes insulin action, and regulates glucose uptake	(20)
Grass carp (*Ctenopharyngodon idellus*), Tuna (*T. tonggol*), Sardinelle (*Sardinella aurita*), Tilapia (*Oreochromis niloticus*)	Peptides, carotenoids, essential fatty acids such as phenylalanine, histidine, tryptophan, methionine	Anticancer properties against colon, breast, ovarian, and antimicrobial	(27)
Tuna (*T. tonggol*), Sardinelle (*S. aurita*), Mackerel fish (*Rastrelliger*), Atlantic salmon (*S. salar*)	Omega-3 fatty acids, DHA, EPA, bioactive peptides, zinc, choline	Restore cognitive damage, prevent cognitive impairment, promote the number of pyramidal cells in the cerebral cortex, and prevent brain cell damage	(12)
Sardinelle (*S. aurita*), Atlantic salmon (*S. salar*), Tuna (*T. tonggol*), Tapra fish (*Opisthopterus tardoore*)	PUFA, DHA, EPA, peptides	Antiobesity blocks; lipid synthesis enzymes inhibit the entry of free fatty acids; in adipocytes, it suppresses lipogenesis, regulates satiety level, and promotes fat oxidation level	(19)
Yellowfin sole (*Limanda aspera*), Salmon (*S. salar*), Tuna (*T. tonggol*), tilapia (*O. niloticus*)	Omega-3 fatty acids, peptides, proteins, chitin, chitosan	Reduce the risk of oxidative stress-related diseases, prevent DNA damage, and suppress the oxidation of linoleic acid	(19)
Sardinelle (*S. aurita*), mackerel fish (*Rastrelliger*), Atlantic salmon (*S. salar*), Snakehead fish (*Channa striata*), Barramundi (*Lates calcarifer*)	Tryptophan, omega-3 PUFA, polyamines, Vitamin D, taurine	The immunomodulatory effect reduces the progression of chronic diseases, regulates the immune cells to fight against viral infections	(25)

DHA, docosahexaenoic acid; PUFA, polyunsaturated fatty acids; EPA, eicosapentaenoic acid.

## Nutritional value of fish

Fish consumption is high due to its exceptional nutritional, delicious taste, and chemical profile. Fish remain the richest source of protein, vitamins, minerals, and healthy fats like omega-3 fatty acids, and contain negligible carbohydrates. According to WHO, the recommended consumption of fish for adults is 340 g/week, for children is 170 g/week, for pregnant women is 340 g/week, and for breastfeeding women is 340 g/week (16). The nutrient components in fish vary from species to species, 100 g of raw fish yields 11.9–20.6 g of protein, 0.3–18.3 g of fat, 60.2 to 85.4 g of moisture content, 0.7–5.3 g of ash, and 267–1020 kJ and may vary from specie to specie (29). A recent study provided a comparison of different marine fish based on proximate composition, such as Rupchanda (*Pampus chinensis*) contains high content of fat, Churi (*Lepturacanthus savala*) contains a high amount of protein and amino acids, and tuna (*Tunnus albacores*) contains high content of lauric acid (30). Malnutrition is a serious nutritional problem in children, and approximately 80% of them are malnourished. The highest value of essential nutrients in different fish species helps to eradicate malnutrition and other chronic nutritional deficiencies (3). The high protein content in fish can potentially reduce the prevalence of protein-energy malnutrition in children. The amount of fatty acids, such as polyunsaturated omega-3 fatty acids (PUFA) and monounsaturated fatty acids (MUFA), reduces the risk of coronary heart disease (31). Fatty acids provide benefits against multiple diseases and enhance quality of life. A significant part of animal protein comes from fish when consumed in adequate quantity in a daily diet (32). In addition to protein, fish also contain major micronutrients such as zinc, calcium, iron, magnesium, phosphorus, and iodine that specifically provide biological properties to vulnerable groups, including children, women, and pregnant women. These nutrients help to meet daily nutritional requirements in the human body and aid in reducing nutritional deficiencies. Thus, enhancing food security and eradicating the burden of malnutrition in public health (33).

## Macronutrients in fish

Numerous studies showed that dietary carbohydrates are present at a minimum value of about < 0.5% and considered zero. Generally, all fish species are excellent sources of macronutrients such as protein and fat, but low in carbohydrates (34, 35). The fat content in fish is categorized into three major categories: lean fish contain < 2.5% fat, medium fatty fish contain 2.5%−6% fat, and fatty fish contain >6%−25% (34). Fish also contain essential polyunsaturated fatty acids, specifically omega-3 fatty acids, omega-6 fatty acids, eicosapentaenoic acid (EPA), and docosahexaenoic acid (DHA) that have essential roles in physiological conditions (36). Fish remain a potential source of animal protein and comprise 15%−24% of the nutritional composition. Fish protein has vast applications in the pharmacological and nutraceutical industries. The by-products of fish, such as fish oil, skin, and bones, are also good animal protein sources and provide functional and biological properties to human health (37). In the nutraceutical industry, fish by-products are used to fortify food and develop nutraceuticals that help treating various human disorders and nutritional deficiencies. The by-products manufactured by fish bones contain adequate essential minerals, including calcium, sodium, potassium, and phosphorus (38).

## Vitamins and minerals in fish

Vitamins comprise a significant part of the nutritional value of different fish, which play a vital role in the chemical processes of the human body (37). Other fish species are good sources of vitamins, such as vitamins A, D, E, and K. Vitamin E plays a vital role in various body functions, including antioxidant properties. Fish species, such as salmon, mackerel, and trout, are enriched with vitamins A and D, which play an important role in bone formation, normal growth, and prevention of vision-related diseases (39). Fish remain a potential source of essential minerals, such as selenium, zinc, iron, iodine, and phosphorus. Selenium and iodine are two potential minerals that significantly affect antioxidation, regulation of body metabolism, and growth and development during childhood (37). The mineral composition of typical fish per 100 g (19) is shown in Table 3.

**Table 3:** Mineral composition of typical fish per 100 g (19, 37).

**Fish Species**	**Minerals per 100 g**									
	**Sodium (mg)**	**Iron (mg)**	**Calcium (mg)**	**Magnes ium (Mg)**	**Potassi um (mg)**	**Zinc (mg)**	**Selenium (mcg)**	**Iodine (mcg)**	**Phosphorus (Mg)**	**Copper (mg)**
Alantic salmon	40–60	0.3–0.8	9–20	27–30	350–450	0.4–0.6	35–50	50–55	220–230	0.04–0.06
Tuna	35–50	0.9–1.5	15–30	35–50	350–450	0.8–1.2	60–95	50–55	280–290	0.05–0.08
Cod	54–60	0.4–0.6	16–20	23–25	413–415	0.5–0.8	32–35	110–115	203–210	0.01–0.03
Macker el	98–101	1.3–1.6	12–15	30–35	400–410	1.0–1.3	40–50	60–65	210–216	0.10–0.15
Tilapia	50–60	0.6–0.8	10–13	20–25	365–370	0.4–0.6	30–40	30–35	175–180	0.05–0.07
Sardines	200–250	2.0–2.9	200–400	35–40	380–450	1.3–1.5	45–65	90–98	480–490	0.25–0.28

## Health benefits of fish and its by-products: antihypertensive

**Figure 1:**
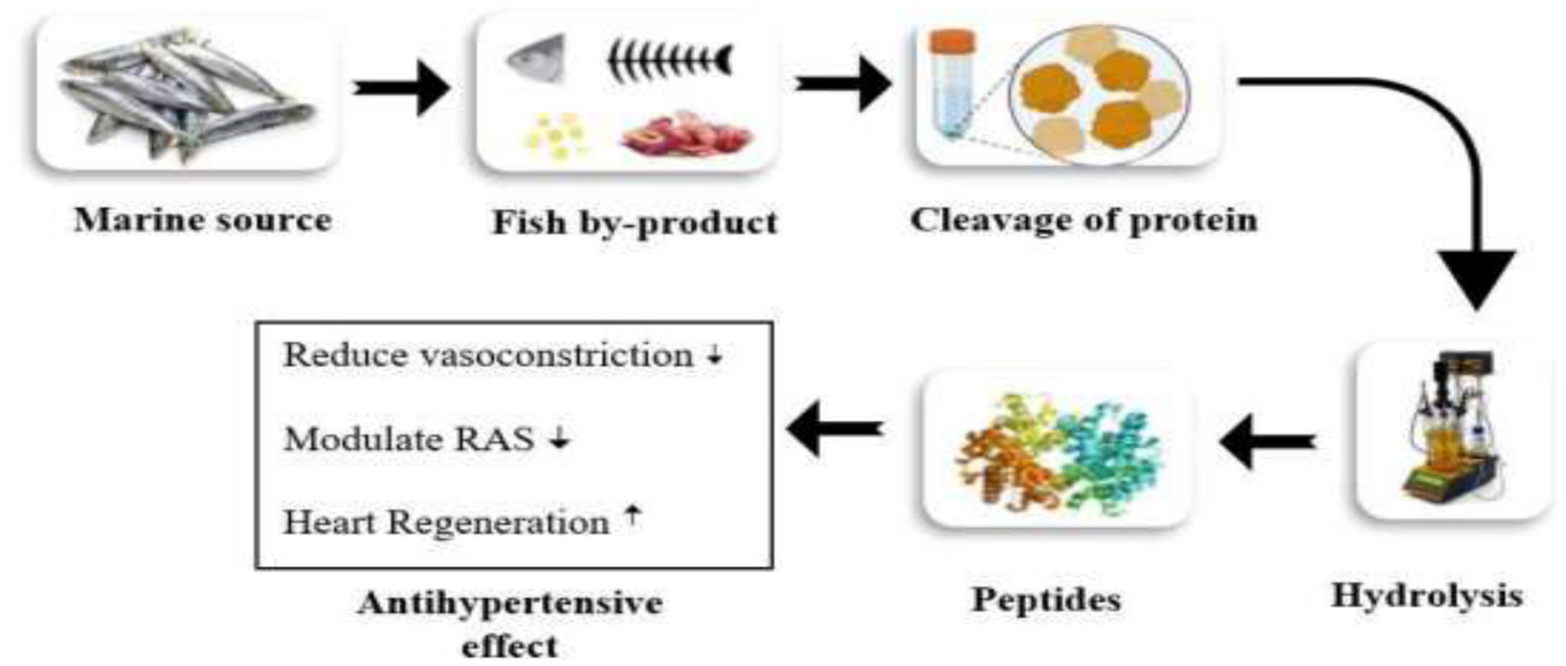
Antihypertensive effects of hydrolysate peptides against hypertension.

Several studies documented that marine fish are enriched with omega-3 polyunsaturated fatty acids, specifically eicosapentaenoic acid (EPA), and docosahexaenoic acid (DHA). The intake of omega-3 fatty acids for an extended period is associated with enhancing endothelial functions by lowering oxidative stress and blood pressure through the production of vasodilators such as nitric oxide (NO) and prostaglandins. The combined effect of DHA and EPA contributes to preventing coronary artery disease (40). Different fish species contain several bioactive compounds with potential biological activities, including antihypertensive properties. The significant antihypertensive effect of fish is due to angiotensin-I-converting enzyme (anti-ACE) biopeptides extracted from various fish proteins such as Salmon (*S. salar*), Tuna, and Grass carp (*C. idellus*). The biopeptides bind with the ACE, suppress its activity, and show a strong antihypertension effect. The Grass carp with a dosage of 100 mg/kg BW showed a significant reduction in systolic blood pressure due to the presence of biopeptides, glycine amino acids, and aspartic acid (41). The by-products of fish, particularly fish oil, a rich source of omega-3 polyunsaturated fatty acids, are mainly attributed to decreased cardiac disorders. Various results of the study showed that the dosages of 0.7 and 1.8 g EPA + DHA/kg/body weight (BW)/day exhibited significant effects against vascular reactivity, stiffness of arteries, blood pressure, and hypertensive status (42). Fish are a rich source of vitamin D, which potentially impacts the renin-angiotensin-aldosterone system (RAS) via inhibiting renin expression. Additionally, vitamin D deficiency contributes to increased blood pressure by activating the fibrotic cascade. A recent study proved that vitamin D is associated with significant antihypertensive activity by reducing systolic blood pressure (43). Various monounsaturated fatty acids help protect from the risk of heart failure. Oleic acid is a monounsaturated fatty acid that protects from cardiovascular insulin resistance by reducing proliferation in smooth muscle cells and improving endothelial dysfunction. Oleic acid showed a significant link with inflammatory markers, antithrombotic potency, and heart failure by inhibiting the aggregation of platelets and plaque stability. Further evidence is required to prove the effect of MUFA against CVD risk factors (44). The hydrolysis of marine protein yields bioactive peptides that significantly show antioxidant, antihypertensive, and other biological activities. The peptides, after ingestion, are absorbed and then enter the bloodstream, where they influence their biological function, such as the antihypertensive effect. The mechanism other than inhibition of ACE involves lowering blood pressure. The bioactive peptides from fish protein cause vasodilation by promoting the production of nitric oxide and regulating the renin-angiotensin system, which prevents the constriction of vessels. The significant effect of fish oil against hypertension (45) is shown in Figure 1.

Fish-derived bioactive compounds help to manage diabetes, obesity, and related metabolic disorders. Fish species contain significant protein, which, upon hydrolysis, yields bioactive peptides with various physiological effects. The protein in marine sources yields peptides that play an important role in lowering the hyperglycemic level by modulating lipid metabolism, insulin secretion, and pancreatic beta cells. Peptides possess the ability to improve insulin sensitivity by suppressing alpha-glucosidase and thus regulate glucose levels (46). Several studies highlight fish as a rich source of essential nutrients that help combat malnutrition and related deficiencies. Several researchers proved that the by-products of fish, such as skin, bones, and fins, possess potential antidiabetic effects. The collagen peptides derived from tilapia (*O. niloticus*) skin showed a significant antihyperglycemic effect via promoting GLP-1, regulating the glucose tolerance, and reducing insulin sensitivity in diabetic rats (47). Several studies demonstrated that the nutritional composition of fish and its by-products contains essential nutrients, including monounsaturated fatty acids and polyunsaturated fatty acids, that have potential antidiabetic effects. Results from a recent study indicated that supplementation of 500 and 1,000 mg/kg BW of fish oil in diabetic rats showed significant antidiabetic action. The antihyperglycemic activity of fish oil showed a significant effect on insulin-sensitivity levels by suppressing relevant insulinotropic actions and improving the islets of Langerhans in the pancreases (48, 49). Various fish species, such as salmon, shark, shellfish, and fish oil, have potential antidiabetic effects. Collagen protein, oligopeptides, gelatin, MUFA, and PUFA present in marine sources such as fish possesses the potential to lower free fatty acids, restore insulin receptors, reduce glycosylated hemoglobin, regulate glucose metabolism, and improve insulin secretion in type-2 diabetes. These nutritional and bioactive components from salmon skin and shark liver provide biological benefits and contribute to industrial, pharmaceutical, and nutraceutical applications (50). Another study revealed that ingesting marine collagen peptides in the human body significantly lowered the glycated hemoglobin (HbA1c) concentrations, improved insulin sensitivity, modulated glucose metabolism, decreased fasting blood glucose concentration, and maintained insulin secretion (51).

### Antihepatotoxic

**Figure 2:**
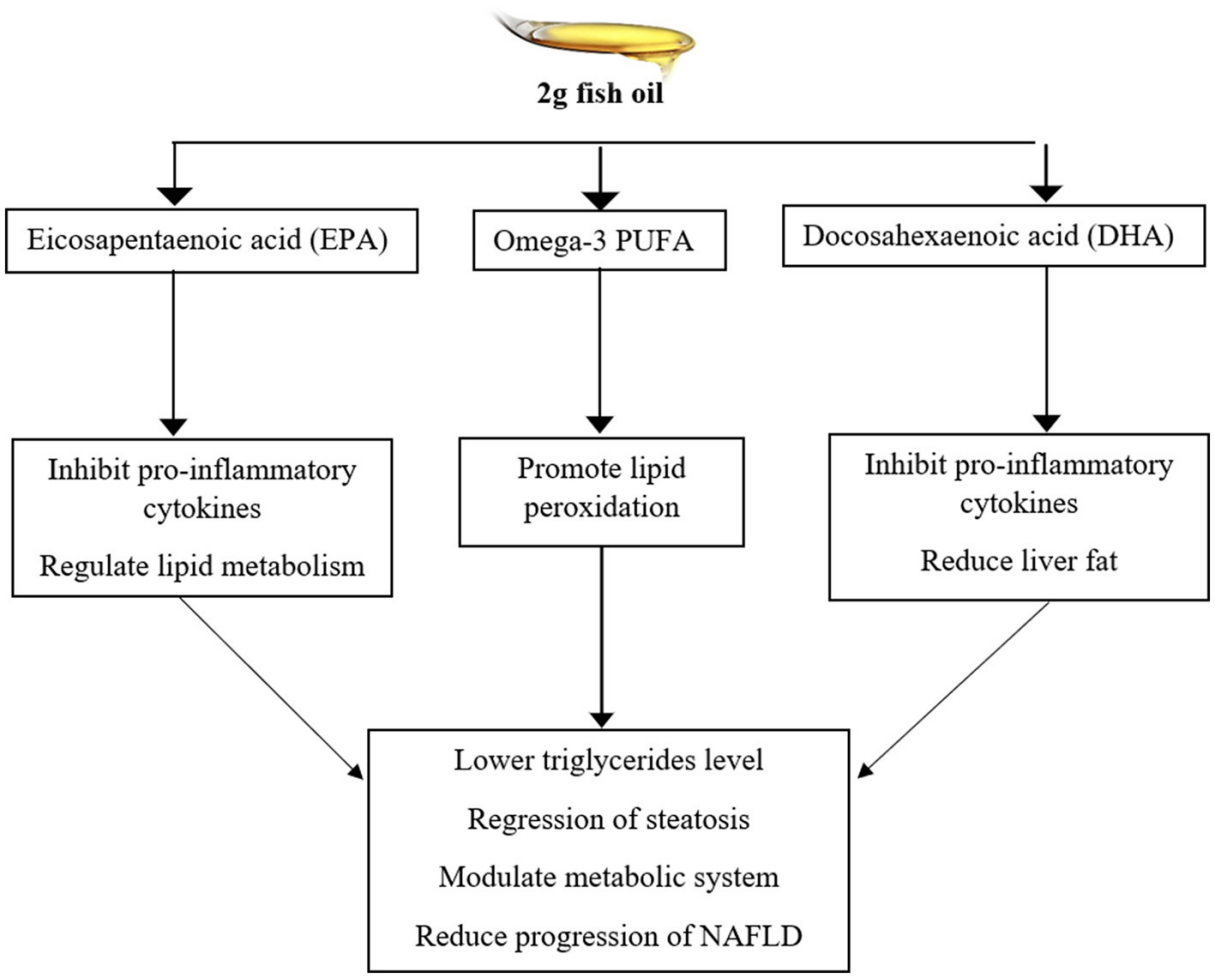
The supplementation of 2-g fish oil showed a significant effect against lipid metabolism and NAFLD.

Various bioactive compounds in food play a vital role in nutraceutical and pharmaceutical applications. Phenolic compounds aid in developing products for treating multiple physiological conditions. Marine sources contain essential bioactive components that possess potential biological activities. The findings showed that fish oil exerted a significant hepatoprotective effect against serum marker enzymes by reducing oxidative stress (52, 53). Fish by-products, particularly fish oil, are used in treating various disorders. Fish oil (FO) is the primary source of antioxidants and omega-3 fatty acids that provide a defense mechanism against free radicals. The results of the study indicated that the treatment of 15% fish oil in hepatotoxicity induced rats exerted a significant effect against liver toxicity by decreasing cell proliferation and improving serum liver enzymes, specifically serum glutamate pyruvate transaminase. Omega-3 fatty acids and omega-6 fatty acids in FO exert hepatoprotective effects by suppressing inflammatory pathways, modulating lipid metabolism, and promoting the β-oxidation of fatty acids (10). Marine organisms are rich sources of essential amino acids and minerals that possess hepatoprotective and pharmacological properties. *Stolephorus commersonnii* (Commerson's anchovy) is reported as a hepatoprotective fish due to the abundance of minerals, particularly unsaturated fatty acids (PUFA and MUFA). The PUFA activates the antifibrotic, antioxidant, and anti-inflammatory mechanisms to prevent liver damage. Antifibrotic mechanism of PUFA decreases liver fibrosis by activating hepatic stellate cells and lowering the deposition of collagen in the liver. The administration of fish extract showed a potential therapeutic effect against drug-induced hepatotoxicity by modifying biochemical markers, including alkaline phosphatase (ALP), serum glutamic pyruvic transaminase (SGPT), and bilirubin (49). Another study demonstrated the effect of fish oil against dyslipidemic rats. The rats were induced with a hypercholesterolemic diet (0.1% cholesterol, egg yolk cholesterol, and 0.5% cholic acid) and divided into groups to identify the effect of a hypocholesterolemia diet with other supplementation for 8 weeks. The group with a hypocholesterolemia diet and 1-mL/day fish oil showed significant results against hepatic lipogenesis that enhanced β-oxidation of fatty acids and improved liver function biomarkers (53). The supplementation of 2-g of fish oil for 6 months exhibits a significant effect against alcoholic fatty liver disease (NAFLD), liver damage. It improves lipids, including serum low-density lipoprotein (LDL) cholesterol, HDL cholesterol, triglycerides, and serum protein. The fish oil inhibited inflammatory cytokines, such as tumor necrosis factor (TNF-α) and interleukin-6 (IL-6) levels in patients with NAFLD and other metabolic disorders. The action mechanism of long-chain fatty acids such as MUFA, DHA, and EPA in fish oil for 6 months of treatment potentially improved the lipid profile by lowering triglycerides, regression of steatosis, very low-density lipoprotein (VLDL) level, and oxidative stress, as mentioned in a previous study (54), is presented in Figure 2.

### Anti-inflammatory

Fish and fish-derived products have therapeutic properties that help mitigate inflammatory diseases. Shellfish are rich in bioactive compounds, including proteins, peptides, and essential amino acids such as histidine, arginine, and glutamine. Shellfish and their derived products exert anti-inflammatory effects via nuclear factor kappa B (NF-κB) and MAPK pathways that activate inflammatory response, decrease cell proliferation, and reduce the expression of pro-inflammatory cytokines (55). The findings of another study reported that oral administration of methanolic extract of *Malapterurus electricus* skin with a dosage of 300 mg/kg BW/day for 7 days showed significant therapeutic benefits against monosodium urate (MSU)-induced arthritic Wistar albino male rats' joints. The extract potentially reduces inflammatory markers, including NF-κB, TNF-α, and decreases ankle swelling by lowering uric acid levels in MSU-induced arthritis due to the anti-inflammatory action mechanism of docosahexaenoic acid, palmitic acid, vaccenic acid, alanine, and 9-octadecenoic (56). The potential nutritional value of fish makes it exceptional among various other fish species. Adequate fish consumption in the daily diet is associated with good quality of life. The content of anti-inflammatory compounds in raw fish, including protein, vitamins, minerals, glutathione peroxidase, polyunsaturated fatty acids, monounsaturated fatty acids, Superoxide dismutase, and peptides, showed potential effects against nitric oxide, pro-inflammatory cytokines, and other inflammatory pathological conditions (57). Oily and lean fish, specifically tuna and salmon, contain 1.5–3.0 g fatty acids, including EPA and DHA, that exert potential effects against concentrations of inflammatory mediators and reduce gene expression of inflammatory cytokines. Protein, marine omega-3 fatty acids, and peptides in fish oil supplementation also reduce the risk of rheumatoid arthritis (RA) and inflammatory bowel diseases (IBD) by suppressing the inflammatory pathways (58). Another study revealed that the administration of omega-3 fatty acids from fish oil significantly exerts antiatherogenic and anti-inflammatory properties by decreasing pro-inflammatory mediators, including leukotrienes, interleukins (ILs), including IL-6, and prostaglandins, which reduces the disease activity in RA (59).

### Antiobesity

**Figure 3:**
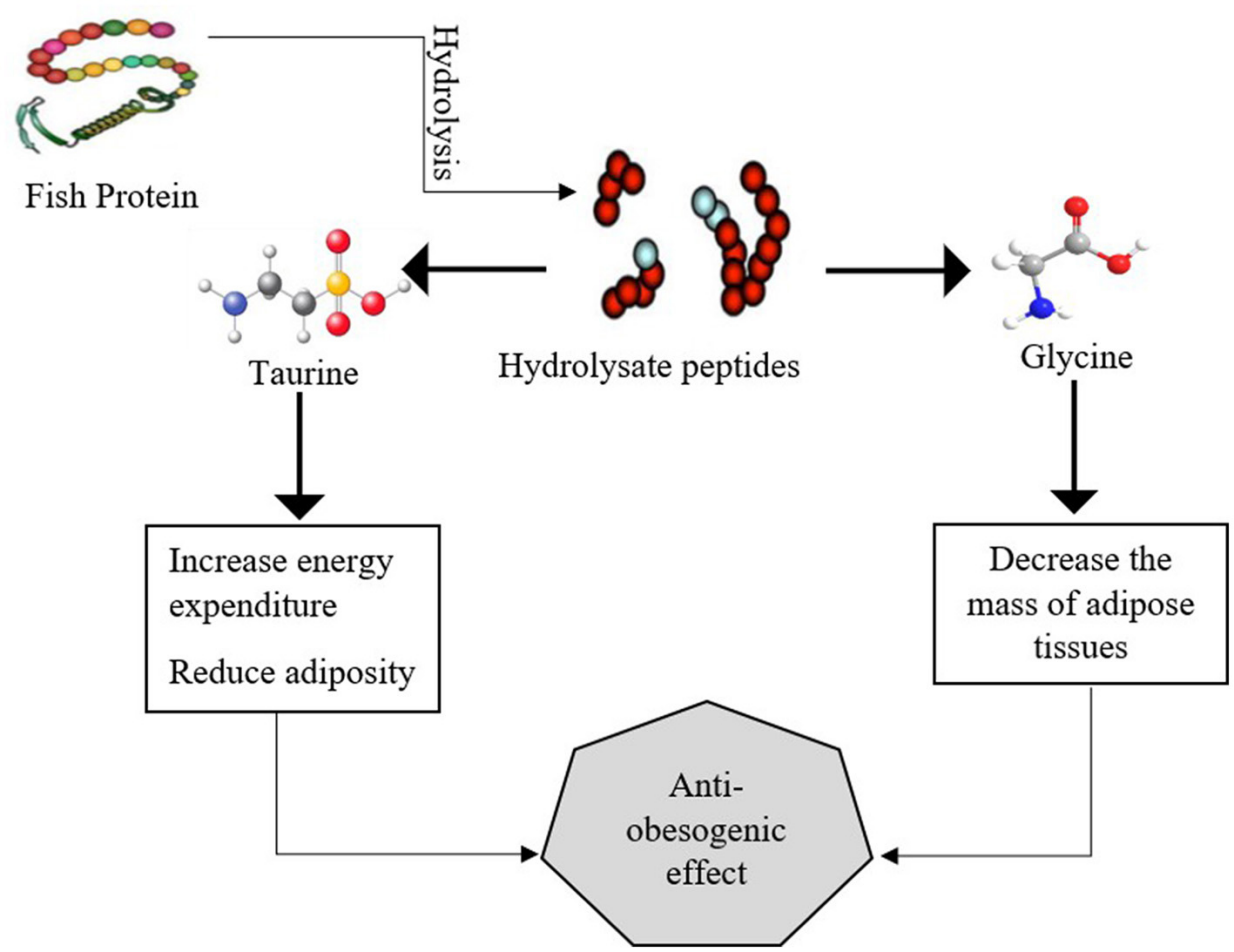
The potential antiobesogenic effect of hydrolysate peptides, glycine, and taurine.

Several fish species, such as salmon, trout, bluefish, tuna, and mackerel, possess significant amounts of peptides, and healthy fatty acids such as PUFA, MUFA, EPA, and DHA. The consumption of 3–4 g omega-3 fatty acids in the daily diet showed a significant association with a healthy metabolic profile. Fish and cod liver oil prevent various obesity-related disorders (60). The results of another experimental study reported that fish oil with a dosage of 12.5 mg/kg BW/day significantly affected obese mice by modulating metabolic pathways, increasing lipid oxidation, decreasing triglycerides, reducing waist circumference, and inhibiting lipogenesis. Omega-3 fatty acids in fish oil play a vital role in suppressing transcription factor SREBP-1 and the ChREBP; these two factors are mainly involved in the synthesis of fatty acids and the regulation of cholesterol. The function of transcription factors SREBP-1 and ChREBP is disturbed in obesity, and instead of regulating the cholesterol level, they enhance the production of triglycerides and promote the obesity-related pathways (61). Marine fish, particularly, fatty fish, such as salmon, sablefish, sardines, fish roe, and tuna, are the primary source of bioactive compounds, polyphenols, omega-3 fatty acids, and essential amino acids, specifically α-linoleic acid (ALA), that play a significant role in reducing obesity-related diseases. These long-chain omega-3 fatty acids potentially decrease adipogenesis, β-oxidation of fatty acids in adipocytes, and suppress adipocyte differentiation and NF-κB pathway in obese mice (62). The by-products of fish, such as skin, bones, and tails, are also excellent sources of dietary protein that exhibit various biological activities. The nutritional properties of fish-hydrolyzed protein also provide vast applications in the field of food industry. Salmon fish provides various pharmaceutical and industrial applications (63). Bioactive peptides in fish protein help to enhance gut absorption and affect energy regulation in the body. These bioactive peptides modulate glucose metabolism, enhance glucose uptake by cells, and promote the oxidation of excess fatty acids. The antiobesogenic effect of marine fish's protein, specifically taurine, glycine, protein, peptides, and omega-3 fatty acids, particularly, EPA and DHA, helps to regulate lipid metabolism, and energy expenditure and modulate endocannabinoid signaling, as presented in a previous study (64), is demonstrated in Figure 3.

### Antioxidant

A wide range of fish species protects from various harmful diseases and improves the quality of life. The action mechanism of nutritional components such as omega-6 fatty acids, omega-3 fatty acids, EPA, and DHA in fish oil exerts a potential influence against oxidative stress. The omega-6 derived metabolites of 5–200 μM concentration play pathological, antiproliferative, and antioxidant effects against inflammatory mediators, including leukotrienes B4 and prostaglandins E2. The findings of the study reported that omega-3 PUFA reduces the progression of GI cancer by regulating the extracellular signal-regulated kinases (ERK) pathway (65). Multiple mechanisms are involved in the suppression and proliferation of tumor cells. The study evaluated the cytotoxic effect of polyunsaturated fatty acids extracted from marine sources such as *S. longiceps* and *Sardinella fimbriata*. EPA in 200 μg/mL extract showed significant antioxidant and anticancer activity by changing gene transcription, inhibiting the growth of tumor cells, suppressing NF-κB activation, and altering prostaglandin production. The study also indicated that EPA potentially reduces toxicity levels in cancerous cells (66). Fish oil supplements comprise abundant amounts of fatty acids, mainly EPA and DHA, that exert antioxidant effects against liver cancer by inhibiting tumor proangiogenic (growth) factors and nuclear signaling pathways (67). The efficacy of omega-3 fatty acids provides beneficial anticancer effects against various types of cancer. The synergistic effect of omega-3 and omega-6 fatty acids inhibits the progression of oxidation in cancer cell lines. The ratio n-6/n-3 PUFA exerts a significant impact on the treatment of colon and liver cancer by reducing cyclooxygenase-2 (COX-2), the number of adenocarcinomas, and TNF-α. The results from another study proved that administration of 1.2 g/kg/day for 7 days exerts a significant effect against colorectal cancer by decreasing cytokines, particularly IL-6 level and serum TNF-α level (68, 69). Various experimental studies demonstrated that fish oil contains selenium that works efficiently in combination with fatty acids against cancer. The synergistic effect of PUFA and selenium significantly reduces cancer progression by suppressing tumor proteins such as TSC1 and TSC2 (tuberous sclerosis complex). The PUFA and selenium showed potential mechanisms against cancer progression by inhibiting the pathways that promote cell proliferation. The pathways involved in cell proliferation include the MAPK pathway, cytokine signaling pathway, and β-Catenin pathway (67).

### Antineurodegenerative

**Figure 4:**
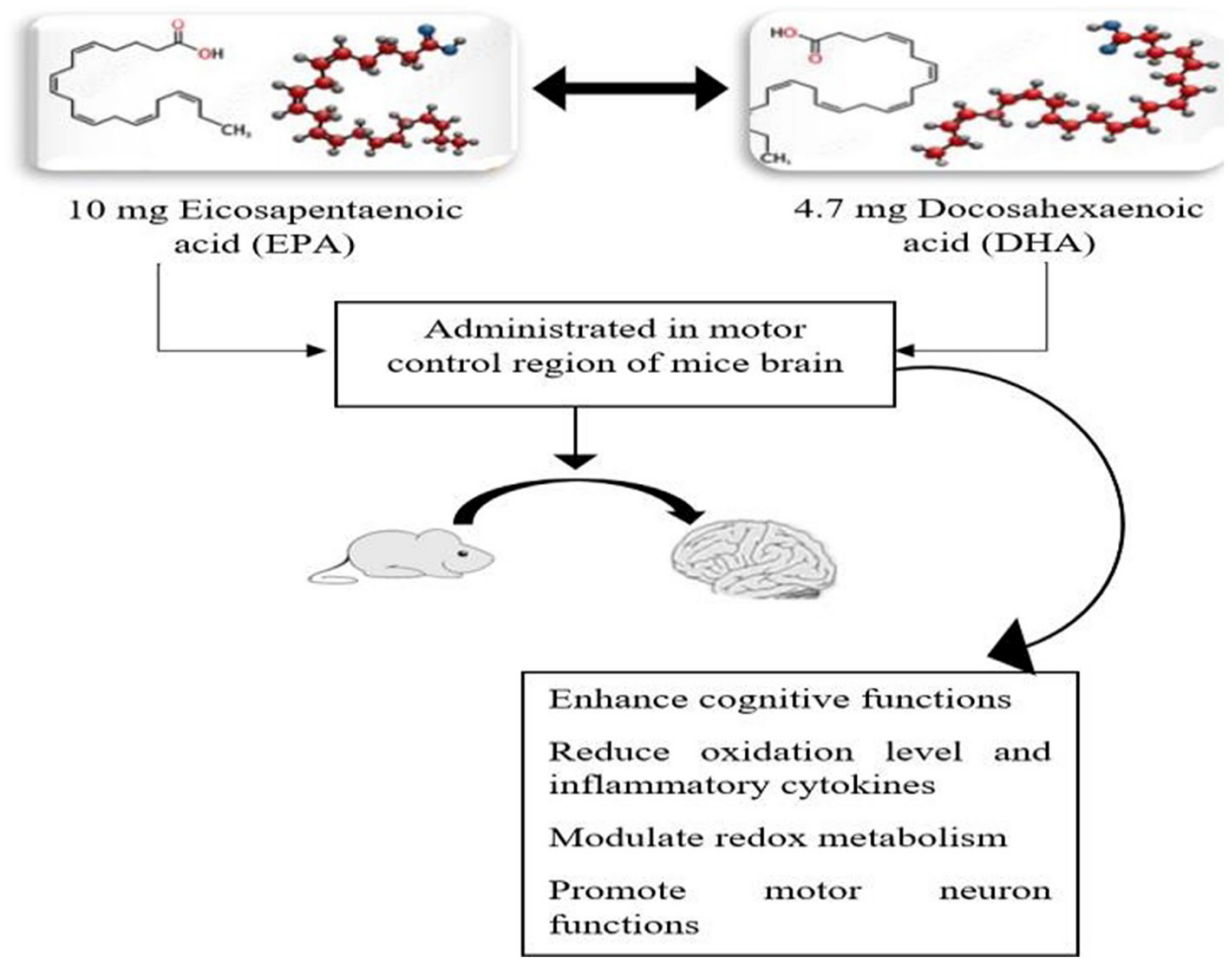
The potential neuroprotective combined effect of supplementation of 10-mg EPA and 4.7-mg DHA on the motor neuron region in mice brain.

Adequate fish consumption in daily life helps to improve brain structure and its functionality. The brain-promoting nutrients in fish and fish oil, such as PUFA, EPA, and DHA, possess the ability to enhance cognitive behavior and thinking skills. The findings of the study also revealed that fish oil supplements prevent incidents of dementia (70). Omega-3 fatty acids, particularly EPA, provide significant neuroprotective effects on the human brain, improve cognitive development, and protect from brain disorders such as Alzheimer's, stroke, dementia, and schizophrenia. The consumption of long-chain fatty acids protects from brain aging. The cerebral blood flow in the brain is enhanced by consuming polyunsaturated fatty acids (DHA) that protect from the progression of neurodegenerative diseases (71, 72). Brain function-promoting nutrients are exclusively present in marine sources. The oral administration of two important polyunsaturated fatty acids, 485-mg EPA and 343-mg DHA, to mice for 21 days examined and evaluated that neuroprotection D-1 (NPD-1) significantly provides a neuroprotective effect and alters the pathogenesis of brain-related diseases (73). In septic patients, impairment and dysfunction in cognition are common after sepsis. The study revealed that consuming omega-3 fatty acids after sepsis in rat brains helps to improve brain-derived neurotrophic factor (BDNF) levels in the hippocampus, reduces inflammatory mediators, and diminishes oxidative markers and cognitive impairment (74). Another study evaluated the neuroprotective effect of fish oil supplementation with a dosage of 20 mg/kg BW/ day for 5 weeks. The finding of the study revealed that FO supplements significantly enhance intelligence, promote learning ability improved long-lasting memory, and reduced SOD levels in the cerebral cortex and hippocampus by regulating BDNF signaling pathways (75, 76). A regular and adequate intake of omega-3 fatty acids derived from seafood and marine sources provides potential neurological benefits. The results of the experimental study indicated that PUFA, specifically DHA, enhances cognitive benefits and provides antioxidants that promote motor neuron functions in the brain. Fish oil supplements were administered at 10-mg EPA/kg BW and 4.7-mg DHA/kg BW for 45 days and evaluated for significant effects against oxidation and enhanced neuron functions. The significant synergistic effect of EPA and DHA in a previous study (77) is presented in Figure 4.

### Antiviral

Aquaculture and marine sources play crucial roles against various types of viruses, including influenza, COVID-19, Herpes simplex virus (HSV), and HIV. The antiviral bioactive compounds in fish oil supplements, such as omega-3 fatty acids, ALA, EPA, DHA, and their derivatives, showed the highest efficacy against viral replication. Protectins, DHA, and its derivatives, specifically D1-protectin and DHA-derived D1-protectin isomer, suppress the replication of the influenza virus by inhibiting the export of mRNA and blocking the viral entry into the nucleus of the influenza virus (78). The pro-resolving mediators derived from omega-3 fatty acids, such as resolvins, lipoxins, and protectins, significantly prevent systemic infection, specifically influenza. These mediators possess the ability to promote immune responses against viruses by inhibiting the proliferation and replication of viral cells. The other constituents in fish have the potential to modulate the immune system and enhance the activity of plasma B cells (79). Another study discussed that fish oil has diverse antiviral and immune regulatory properties to inhibit SARS-CoV-2 infection. PUFA in fish oil contributes to antiviral activities against various viruses by breaking and modulating the viral cell membrane, resulting in viral cell lysis. These essential fatty acids also protect against various bacteria, including Staphylococcus epidermidis and Staphylococcus aureus, by inhibiting biofilm formation (80). Omega-3 fatty acids, particularly DHA, EPA, ALA, and AA, provide an immunological defense mechanism against viral replication and infection of SARS-CoV by inhibiting the gateway of the virus, Angiotensin-converting enzyme-2 (ACE2). Salmon and sablefish showed potential positive effects against COVID-19 severity (81). The largest content of fish nutrients comprises essential fatty acids that provide several health benefits and protect from various human ailments. Immunoregulatory mechanisms of essential fatty acids, including eicosapentaenoic acid (EPA) and docosahexaenoic acid (DHA) possess the ability to inhibit the massive secretion of cytokines involved in the pathogenesis of COVID-19. Another study in mice discussed that adequate consumption of omega-3 fatty acids in the diet reduces the secretion of IFN-gamma and thus protects the immune system (79).

### Toxicity of fish

Various toxic chemicals come from different sources into rivers, lakes, and streams, contaminating the surface water. The main harmful and toxic substances in marine sources are dieldrin, polychlorinated biphenyls (PCBs), dioxin, and heptachlor. These harmful chemicals accumulate in the fatty tissue of a few fish, and consuming such contaminated fish causes multiple health disorders in the human body, such as cancer (82). Another study discussed that samples collected from the Tigris River of different fish species, including Grass carp, *Cyprinus carpio*, and Liza abu, showed that gills, lungs, and other fish parts were contaminated with lead, copper, and zinc. Heavy metals such as Cu, Zn, and Pb cause extreme toxicity and mutagenesis in the human body. The acceptable levels of zinc, copper, and lead in fish are 3, 2, and 2 mg/kg, respectively (83). Fish are more susceptible to hazardous chemicals and toxic contaminants in marine sources, specifically microplastics. The ingestion of microplastics by fish in aquatic ecosystems and the consumption of such contaminated fish influence the human body adversely. Microplastics negatively affect gut health, brain function, inflammation, and oxidation in the human body. The pathogenic microorganisms in microplastics also affect negatively aquatic food webs (84). The findings of another study discussed that fish are also contaminated with pesticides, including pyrethroids, in aquatic environments. Pyrethroids promote oxidative stress and physical damage, and increase ROS in fish (85, 86).

## Conclusion

The diverse nutritional profile and bioactive compounds found in various fish species and their by-products offer considerable therapeutic potential, significantly benefiting both the fields of nutrition and the food industry. The increased processing of fish and its by-products plays a critical role in combating food insecurity and preventing chronic diseases. As global demand for fish continues to rise, there is a pressing need to improve production techniques, aquaculture environments, and the sustainable processing of by-products. Quality assurance must remain a top priority to mitigate toxicity and health risks associated with fish consumption. A growing demand to enhance advancements in precision aquaculture, genetic improvement of fish species, and biotechnology for extracting bioactive compounds holds great promise. The improvement in aquaculture will also promote the local economies. Adopting digital tools, such as artificial intelligence and blockchain, can enhance traceability and safety in the fish supply chain, while circular economy models can reduce waste and promote sustainability. There is a pressing need to utilize these advancements in fishing communities to promote global nutrition and environmental health. Furthermore, international collaborations and robust marine conservation policies will be essential to protect aquatic ecosystems and ensure the long-term viability of marine resources.
